# The Cytolethal Distending Toxin Contributes to Microbial Virulence and Disease Pathogenesis by Acting As a Tri-Perditious Toxin

**DOI:** 10.3389/fcimb.2016.00168

**Published:** 2016-12-05

**Authors:** Monika D. Scuron, Kathleen Boesze-Battaglia, Mensur Dlakić, Bruce J. Shenker

**Affiliations:** ^1^Department of Pathology, School of Dental Medicine, University of PennsylvaniaPhiladelphia, PA, USA; ^2^Department of Biochemistry, School of Dental Medicine, University of PennsylvaniaPhiladelphia, PA, USA; ^3^Department of Microbiology and Immunology, Montana State UniversityBozeman, MT, USA

**Keywords:** toxin, virulence, PI-3 kinase, inflammation, epithelial cells, lymphocytes, macrophages

## Abstract

This review summarizes the current status and recent advances in our understanding of the role that the cytolethal distending toxin (Cdt) plays as a virulence factor in promoting disease by toxin-producing pathogens. A major focus of this review is on the relationship between structure and function of the individual subunits that comprise the AB_2_ Cdt holotoxin. In particular, we concentrate on the molecular mechanisms that characterize this toxin and which account for the ability of Cdt to intoxicate multiple cell types by utilizing a ubiquitous binding partner on the cell membrane. Furthermore, we propose a paradigm shift for the molecular mode of action by which the active Cdt subunit, CdtB, is able to block a key signaling cascade and thereby lead to outcomes based upon programming and the role of the phosphatidylinositol 3-kinase (PI-3K) in a variety of cells. Based upon the collective Cdt literature, we now propose that Cdt is a unique and potent virulence factor capable of acting as a tri-perditious toxin that impairs host defenses by: (1) disrupting epithelial barriers; (2) suppressing acquired immunity; (3) promoting pro-inflammatory responses. Thus, Cdt plays a key role in facilitating the early stages of infection and the later stages of disease progression by contributing to persistence and impairing host elimination.

## Introduction

The cytolethal distending toxin (Cdt) was first described by Johnson and Lior (1988a,b) as a heat-labile toxin present in culture filtrates obtained from clinical isolates of *Campylobacter* spp. (including *C. jejuni, C. coli, C. fetus, C. lari*), *Escherichia coli* and *Shigella dysenteriae*. Specifically, Cdt-containing filtrates were reported to adversely affect CHO cells, Vero cells, HeLa cells, and Hep-2 cells by inducing a progressive distention over 96 h and eventually leading to loss of cell vitality between 96 and 120 h. Interestingly, the murine adrenal tumor cell line, Y-1, was not susceptible to Cdt toxicity. This was a key observation as Y-1 cells were commonly used to assess the classic heat-labile toxin (LT), thereby allowing these investigators to proclaim the discovery of a new and unique toxin. To date there has been much progress on our understanding of Cdts since the report of these seminal observations. For instance, Cdts are now known to represent a conserved and highly distributed family of putative virulence factors produced by a diverse group of more than 30 γ- and ε-Proteobacteria which are responsible for chronic infection and inflammatory disease typically affecting mucocutaneous tissue (summarized in Table 1). Human pathogens that produce Cdt include: an oral pathogen, a genital pathogen responsible for sexually transmitted chancroid, gastric pathogens, and carcinogenic pathogens; these include both human and animal pathogens (Whitehouse et al., 1998; Shenker et al., 1999; Lara-Tejero and Galán, 2001; Haghjoo and Galán, 2004; Thelastam and Frisan, 2004; Young et al., 2004; Ge et al., 2005, 2007; Gargi et al., 2012; DiRienzo, 2014). Moreover, regardless of microbial source, all Cdts cause similar effects on a range of proliferating experimental target cells: cell cycle arrest and eventual cell death mediated by activation of the apoptotic cascade (Jinadasa et al., 2011). More recent observations suggest that Cdt is capable of inducing functional alterations in the absence of cell death in non-proliferating populations (Shenker et al., 2010, 2014).

**Table 1:** **Cdt Producing bacteria^a^**.

**Bacterium**	**CDT**	**Host**	**Niche**	**Cell type affected**				**References**
				**Epithelium**	**Macrophages**	**Lymphocytes**	**Other**	
**CLASS *Epsilonproteobacteria***								
**FAMILY *Campylobacteriaceae*** ***Campylobacter* SPECIES**								
*C. coli*	CcolCDT	Human, Non-human primates, Cattle, Sheep, Pig, Chicken	Intestinal mucosa	HeLa, CHO, Vero, Y-1 (mouse adrenal gland epithelial cells)				Pickett et al., 1996; Bang et al., 2003; Dassanayake et al., 2005b; Fouts et al., 2005
*C. fetus* subsp. *fetus*	CfetCDT	Human, Cattle, Sheep	Intestinal mucosa, Urogenital mucosa	HeLa				Johnson and Lior, 1988a; Ohya et al., 1993; Pickett et al., 1996
*C. fetus* subsp. *venerealis*	CvenCDT	Human, Cattle	Intestinal mucosa, Urogenital mucosa	HeLa				Asakura et al., 2008; Moolhuijzen et al., 2009
*C. hyointestinalis*	ChyoCDT	Human, Cattle, Pig	Intestinal mucosa	HeLa				Gebhart et al., 1983; Edmonds et al., 1987; Moolhuijzen et al., 2009
*C. jejuni*	CjejCDT	Human, Non-human primates, Cattle, Sheep, Pig, Dog, Cat, Ferret, Chicken	Intestinal mucosa, Liver	HeLa, Caco-2, Henle-407, CHO, Vero, Y-1 (mouse adrenal gland epithelial cells)	28SC human monocytic cell line		COS-1 fibroblast-like cells	Fox et al., 1987; Johnson and Lior, 1988a; Whitehouse et al., 1998; Lara-Tejero and Galán, 2000; Bang et al., 2003; Fouts et al., 2005; Hickey et al., 2005; Young and Mansfield, 2005; Young et al., 2007; Sahin et al., 2008
*C. lanienae*	ClanCDT	Sheep	Intestinal mucosa					Acik et al., 2013
*C. lari* (formerly *laridis*)	ClarCDT	Human, Sheep	Intestinal mucosa	HeLa, CHO, Vero, Y-1 (mouse adrenal gland epithelial cells)				Johnson and Lior, 1988a; Pickett et al., 1996; Fouts et al., 2005; Shigematsu et al., 2006; Acik et al., 2013
*C. upsaliensis*	CupsCDT	Human, Pig, Dog, Cat, Chicken	Intestinal mucosa	HeLa				Pickett et al., 1996; Mooney et al., 2001; Fouts et al., 2005
**FAMILY *Helicobacteriaceae*** ***Enterohepatic Helicobacter* SPECIES**								
*Hel. bilis*	HbilCDT	Laboratory mice, Dog	Intestinal mucosa, Biliary mucosa	HeLa				Dusek et al., 1994; Chien et al., 2000; Kostia et al., 2003; Fox et al., 2004b; Fox, 2007
*Hel. canis*	HcanCDT	Human, Dog	Intestinal mucosa, Liver	HeLa				Fox et al., 1996; Chien et al., 2000; Leemann et al., 2006
*Hel. cinaedi*	HcinCDT	Human, Non-human primates, Laboratory mice	Intestinal mucosa, Liver	HeLa				Fernandez et al., 2002; Taylor et al., 2003; Shen et al., 2009
*Hel. hepaticus*	HhepCDT	Laboratory mice	Intestinal mucosa	HeLa, Caco-2. HT-29, HCA-7, INT-407				Young et al., 2000a,b; Liyanage et al., 2013; Péré-Védrenne et al., 2016
*Hel. marmotae*	HmarCDT	Woodchucks	Liver	HeLa				Chien et al., 2000; Taylor et al., 2003
*Hel. mastomyrinus*	HmasCDT	Laboratory mice, Mastomys	Intestinal mucosa, Liver	HeLa				Shen et al., 2005
*Hel. pullorum*	HpulCDT	Human, Chicken, Laboratory mice	Intestinal mucosa	Caco-2. HT-29, HCA-7, HEK-293, HeLa				Young et al., 2000b; Ceelen et al., 2006b; Boutin et al., 2010; Péré-Védrenne et al., 2016
*Hel. winghamensis*	HwinCDT	Human	Intestinal mucosa					Melito et al., 2001
**CLASS *Gammaproteobacteria***								
**FAMILY *Enterobacteriaceae*** ***Escherichia* SPECIES**								
*E. albertii*	EalbCdtB-I	Birds	Intestinal mucosa					Oaks et al., 2010
*E. albertii*	EalbCdtB-II	Human, Birds	Intestinal mucosa					Oaks et al., 2010
*E. albertii*	EalbCdtB-III	Human, Birds	Intestinal mucosa	HeLa				Hyma et al., 2005; Oaks et al., 2010
*E. albertii*	EalbCdtB-IV	Birds	Intestinal mucosa					Wang et al., 2016
*E. albertii*	EalbCdtB-V	Human, Birds	Intestinal mucosa	HeLa				Hyma et al., 2005; Oaks et al., 2010
Enteropathogenic/extraintestinal pathogenic/avian pathogenic *E. coli*	EcolCdtB-I	Human, Chicken	Intestinal and urogenital mucosa	Human colonic epithelial cells, HeLa, HEp-2, CHO, Vero				Asakura et al., 2007; Johnson et al., 2007; Graillot et al., 2016
Enteropathogenic *E. coli*	EcolCdtB-II	Human	Intestinal mucosa	CHO				Pickett et al., 1994; Pickett and Whitehouse, 1999; Bielaszewska et al., 2005
Enteropathogenic/ extraintestinal pathogenic/ necrotoxigenic *E. coli*	EcolCdtB-III	Human, Cattle	Intestinal mucosa	HeLa, CHO				Pérès et al., 1997; Pickett and Whitehouse, 1999; Toth et al., 2003; Bielaszewska et al., 2004; Johnson et al., 2010
Enteropathogenic/extraintestinal pathogenic/necrotoxigenic *E. coli*	EcolCdtB-IV	Human, Pig, Chicken	Intestinal and urogenital mucosa	HeLa, CHO				Toth et al., 2003, 2009; Bielaszewska et al., 2005
Enterohaemorrhagic/Shiga toxin-producing *E. coli*	EcolCdtB-V	Human, Cattle	Intestinal mucosa	HeLa, CHO			HUVEC, HBMEC endothelial cells	Bielaszewska et al., 2004, 2005; Cadona et al., 2013; Svab et al., 2013; Taieb et al., 2015
***Providencia* SPECIES**								
*P. alcalifaciens*	PalcCDT	Human	Intestinal mucosa	CHO				Shima et al., 2012
***Shigella* SPECIES**								
*Shig. boydii* serotype 13	SboyCDT	Human	Intestinal mucosa	HeLa				Johnson and Lior, 1987; Hyma et al., 2005
*Shig. dysenteriae*	SdysCDT	Human	Intestinal mucosa	CHO, Mouse epithelial cells of the descending colon				Johnson and Lior, 1987; Okuda et al., 1997
***Salmonella* SPECIES**								
*S. enterica* serotype Typhi	StypCdtB	Human	Intestinal mucosa	Henle-407				Haghjoo and Galán, 2004
*S. enterica* serovar Brandenburg	SbraCdtB	Birds	Intestinal mucosa					Skyberg et al., 2006
*S. enterica* serovar Bredeney	SbreCdtB	Human, Birds	Intestinal mucosa, Blood					Skyberg et al., 2006; Suez et al., 2013
*S. enterica* serovar Javiana	SjavCdtB	Human	Intestinal mucosa	HeLa				Mezal et al., 2014
*S. enterica* serovar Montevideo	SmonCdtB	Human	Intestinal mucosa, Blood					Suez et al., 2013
*S. enterica* serovar Schwarzengrund	SschCdtB	Humna, Birds	Intestinal mucosa, Blood					Skyberg et al., 2006; Suez et al., 2013
*S. enterica* serovar Typhimurium	StyphimCdtB	Human, Poultry, Pig, Cattle	Intestinal mucosa	HeLa				Figueiredo et al., 2015
*S. enterica* serovar 9,12:l, v:-	S9,12CdtB	Human	Intestinal mucosa, Blood					Suez et al., 2013
**FAMILY *Pasteurellaceae*** ***Aggregatibacter* SPECIES**								
*Aggregatibacter* (formerly *Actinobacillus*) *actinomycetemcomitans*	AactCDT	Human	Periodontal pocket, Gingival sulcus, Dental plaque	Human gingival epithelial cells, KB, Ca9-22, HeLa, HEp-2, CHO	Human monocytes, U937, Raw 264.7 murine macrophages	Human PBMC, Jurkat, Molt-4, HS-72	Human gingival fibroblasts, Human periodontal ligament cells	Sreenivasan et al., 1991; Ohguchi et al., 1998; Sugai et al., 1998; Shenker et al., 1999; Akifusa et al., 2001; Yamamoto et al., 2004; Belibasakis et al., 2005a,b; Kang et al., 2005; Ohara et al., 2008; Rabin et al., 2009; Shenker et al., 2010; Ando-Suguimoto et al., 2014; Shenker et al., 2014
***Avibacterium* SPECIES**								
*Avibacterium* (formerly *Haemophilus*) *paragallinarum*	AparCDT	Chicken	Respiratory tract	HeLa			DF-1 (chicken embryo fibroblasts)	Chen et al., 2014
***Haemophilus* SPECIES**								
*Haemophilus ducreyi*	HducCDT	Human	External genitalia	HeLa, HEp-2, INT-407, Caco-2, A549, HaCat, CHO	Human monocytes, THP-1, U937	Human B-cells, BL-41 lymphoma, granulocytes	Human foreskin, human embryonic lung, BJ, IMR-90, WI-38 and Don fibroblasts, U2-OS (bone), HMVEC-d, EGM-2MW and HUVEC endothelial cells	Purvén and Lagergard, 1992; Ahmed et al., 2001; Svensson et al., 2001; Blazkova et al., 2010; Gargi et al., 2013; Dixon et al., 2015
*Haemophilus parasuis*	HparCDT	Pig	Upper respiratory mucosa					Yue et al., 2009

a *The expression of cdtB was confirmed in Salmonella serotypes listed in this table. However, the authors recognize the fact that up to date more than 40 non-typhoidal Salmonella serotypes were shown to encode cdtB (den Bakker et al., 2011)*.

Cdts are encoded by three genes, designated *cdtA, cdtB*, and *cdtC*, which are arranged as an operon which encodes three polypeptides which have been designated CdtA, CdtB, and CdtC with apparent molecular masses of 23–30, 28–32, and 19–20 kDa, respectively; these Cdt subunits form a heterotrimeric holotoxin (Pickett and Whitehouse, 1999; Shenker et al., 1999, 2000, 2001; De Rycke and Oswald, 2001; Thelastam and Frisan, 2004). Cdt toxin subunits are encoded by three marginally overlapping chromosomal genes *cdtA, cdtB*, and *cdtC* (Pickett et al., 1994; Scott and Kaper, 1994; Okuda et al., 1995). However, *Escherichia coli cdtB*-III gene cluster is located on a transferable plasmid pVir (Pérès et al., 1997). In contrast to all other bacteria expressing Cdt, *Salmonella enterica* serotype Typhi (*S*. Typhi) lack genes encoding CdtA and CdtC (Haghjoo and Galán, 2004; Skyberg et al., 2006; Suez et al., 2013; Mezal et al., 2014; Figueiredo et al., 2015).

Although all Cdts exhibit similar activity, their amino acid sequences diverged among different bacterial species (Nesic et al., 2004). The CdtB sequences are more conserved among the Cdt-producing bacteria while the CdtA and CdtC subunits display a higher degree of variability (Cortes-Bratti et al., 2001; Lara-Tejero and Galán, 2001; Smith and Bayles, 2006). Bacteria from *Escherichia* genus are unique in the fact that five types of Cdts, termed EcolCdtB-I, EcolCdtB-II, EcolCdtB-III, EcolCdtB-IV, and EcolCdtB-V have been characterized so far based on CdtB subunit divergence. *C. jejuni* Cdt displays a relatively high amino acid sequence similarity to *E. coli* toxins and interacts with host cells in a similar way (Eshraghi et al., 2010). The Cdts from *Haemophilus ducreyi* and *Aggregatibacter actinomycetemcomitans* share only limited amino acid sequence homology with *E. coli* Cdt and were shown to have different requirements for intoxication and intracellular trafficking in host cells (Eshraghi et al., 2014). The *cdtB* genes from seven *Campylobacter* species are conserved and can be amplified using common primers (Kamei et al., 2014).

**Figure 1:**
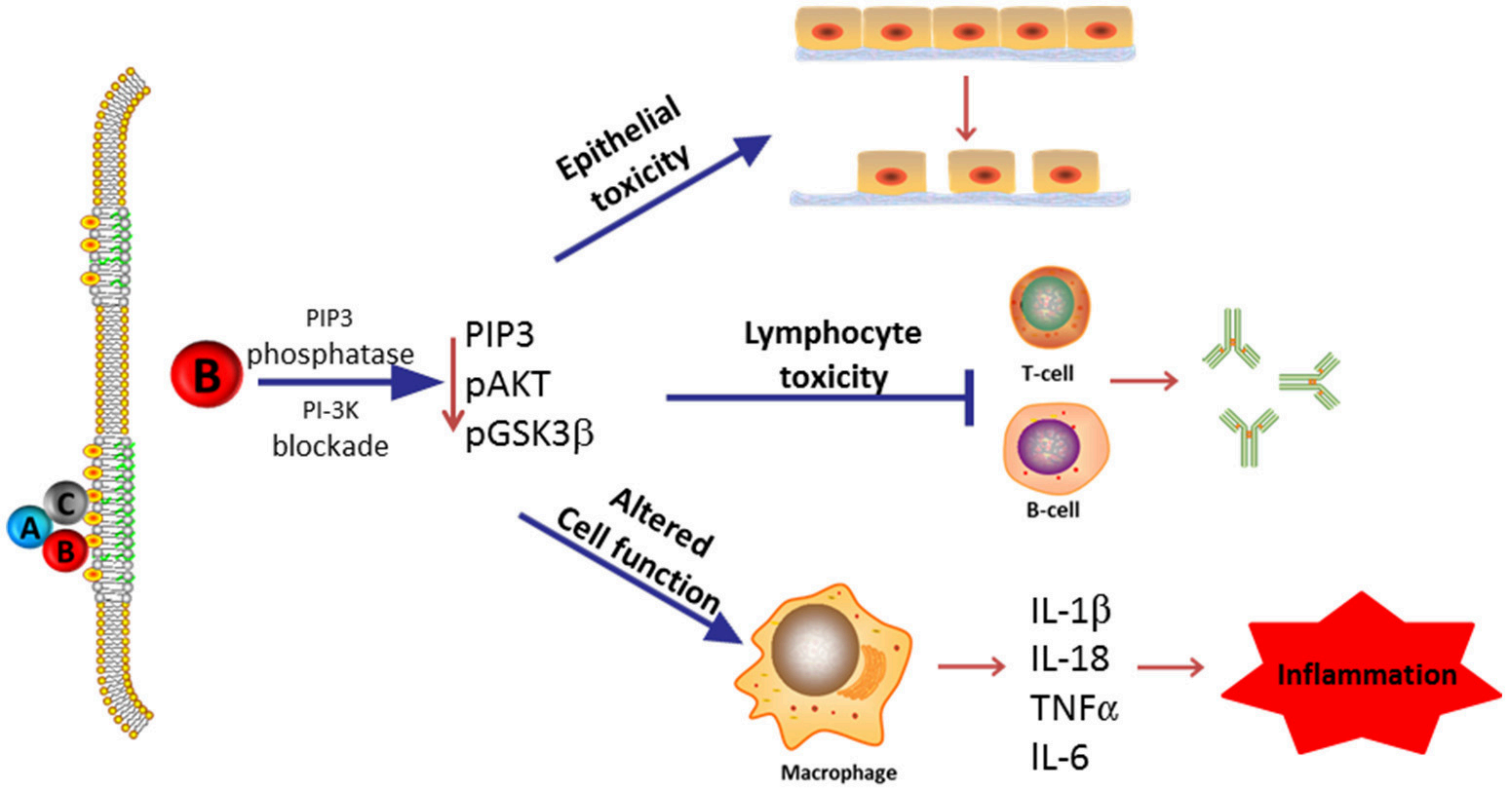
**Overview of Cdt's role as a virulence factor**. Most Cdts are heterotrimeric AB_2_ toxins that are capable of affecting multiple cell types due to the ability of the binding (B) subunits to bind to cells via a common membrane moiety, cholesterol. Furthermore, the active (A) subunit, CdtB, functions as a PIP3 phosphatase and is therefore capable of inducing a blockade of a universally employed signaling cascade, PI-3K. We now propose that in its capacity as a virulence factor, Cdt functions as a tri-perditious toxin that is capable of impairing host defense in three ways: (1) induce cell cycle arrest and apoptosis in epithelial cells thereby altering epithelial barriers and facilitating infection by Cdt-producing pathogens; (2) induce cell cycle arrest and apoptosis in lymphocytes thereby impairing acquired immunity and promoting persistent infection; and (3) altering macrophage function leading to a pro-inflammatory response as a result of increased cytokine synthesis and inflammasome activation. Please note that we have not excluded the possibility that some Cdts and/or some cells may under some conditions be intoxicated as a result of CdtB associated DNase activity.

In this review, we summarize our current understanding of the functional role of the subunits that comprise the Cdt holotoxin and, in particular, discuss the molecular mechanisms that underlay Cdt association with host cells and which contribute to the key events leading to toxicity. As Cdt has profound effects on multiple types of host cells, we discuss the evidence supporting a role for Cdt as a virulence factor. Specifically, we propose that Cdt is a unique and potent virulence factor capable of acting as a tri-perditious toxin that impairs host defenses by: (1) disrupting epithelial barriers; (2) suppressing acquired immunity; (3) promoting pro-inflammatory responses (summarized in Figure 1).

## Cdt subunit structure and function

### Cdt subunits responsible for cell association

It is generally accepted that all three Cdt subunits, CdtA, CdtB, and CdtC, are required to achieve maximal toxic activity, regardless of the target cell. Thus, the holotoxin is believed to function as an AB_2_ toxin where the cell binding unit (B) is responsible for toxin binding to the cell surface and delivery of the active subunit (A) to intracellular compartments. In the context of Cdt, binding activity is considered to be the result of cooperative activities of both the CdtA and CdtC subunits (reviewed in Gargi et al., 2012). Moreover, it has been proposed that these subunits share structural homology with lectin-like proteins and further, that fucose moieties might be involved in toxin association with the cell surface (Nesic et al., 2004; McSweeney and Dreyfus, 2005). In other studies, glycosphingolipids have been implicated as possible binding sites as inhibitors of glycosphingolipid synthesis reduce Cdt intoxication (Mise et al., 2005). It should be pointed out that in a more recent study in which Cdts from different microbial species were studied simultaneously, toxin-host interactions were shown not to depend upon fucosylated structures as well as N- and O-glycans (Eshraghi et al., 2010). These authors did, however, observe that several Cdts, including toxin prepared from *A. actinomycetemcomitans, C. jejuni*, and *H. ducreyi*, utilized membrane cholesterol; these results corroborated the findings of several other investigators (Guerra et al., 2005; Boesze-Battaglia et al., 2009; Zhou et al., 2012; Lai et al., 2013, 2015).

The ability of Cdt to specifically bind to cholesterol has been demonstrated utilizing both model membranes as well as live cells including both lymphocytes and macrophages (Boesze-Battaglia et al., 2006, 2009; Shenker et al., 2014). Moreover, cholesterol specific binding was shown to depend upon an amino acid sequence, the cholesterol recognition amino acid consensus sequence (CRAC), encoded within the CdtC subunit (Boesze-Battaglia et al., 2009). The CRAC site has been defined as: -L/V-(X_1−5_)-Y-(X_1−5_)-R/K- where X_1−5_ represents 1–5 residues of any amino acid. Furthermore, Boesze-Battaglia (Boesze-Battaglia et al., 2009) have demonstrated not only the presence of this site within the *A. actinomycetemcomitans* CdtC subunit, -^68^LIDYKGK^74^-, but also that mutation of key CRAC residues results in reduced toxin binding, CdtB internalization, and toxicity (Boesze-Battaglia et al., 2009). More recently, a similar site has been identified for the CdtB subunit of the *A. actinomycetemcomitans* toxin (Boesze-Battaglia et al., 2015) and is the focus of other papers in this section. It is also noteworthy that the presence of CRAC sites and utilization of cholesterol specific binding has been demonstrated for several proteins including the bacterial leukotoxin derived from *A. actinomycetemcomitans*, gp41 a transmembrane protein found in HIV, caveolin, apolipoproteins, benzodiazepine, G-protein coupled receptors, Ca^++^- and voltage-gated K^+^ channels as well as the translocator protein, TSPO (Li and Papadopoulos, 1998; Vincent et al., 2002; Epand et al., 2005; Jamin et al., 2005; Jafurulla et al., 2011; Oddi et al., 2011; Singh et al., 2012; Brown et al., 2013; Lecanu et al., 2013).

It should be noted that not all investigators have observed that Cdts are dependent upon cholesterol. For instance, cholesterol loading experiments failed to alter cell susceptibility to *C. jejuni* (Eshraghi et al., 2010). Other investigators have reported that cholesterol depletion did not alter *A. actinomycetemcomtans* Cdt internalization (Damek-Poprawa et al., 2012). These findings are difficult to assess as the experimental protocol and/or cells utilized varied. For example, in some instances long exposure times to the cholesterol depleting agent, methyl-β-cyclodextrin, were employed; this has the risk of altering cell vitality. Furthermore, cholesterol repletion was not employed as a control to demonstrate both cholesterol specificity and verify that viability was not altered.

In addition to binding to cholesterol, it has been demonstrated that the Cdt holotoxin associates with cholesterol rich membrane microdomains, as demonstrated by co-localization with GM1 (Boesze-Battaglia et al., 2006). Disruption of lipid rafts by cholesterol depletion reduces toxin binding, CdtB internalization and cell susceptibility to Cdt intoxication (Boesze-Battaglia et al., 2009). Association of Cdt with lipid rafts may be critical to toxicity for several reasons. First, lipid microdomains have been shown to serve as a site of attachment for pathogens and provide a means to concentrate receptors for toxins and thereby increase binding affinity. Second, lipid microdomains play a key role in triggering internalization and may provide a means of entry for the CdtB subunit. Finally, lipid microdomains have been shown to also serve as signaling platforms; this would put the catalytic subunit, CdtB, in close proximity to signaling cascades thereby facilitating their hijacking and involvement in mediating toxicity. It should be noted that Cdt association with lipid microdomains and its significance with respect to toxin binding, internalization and molecular mode of action are a topic of other publications in this topic area (Boesze-Battaglia et al., 2016; Lai et al., 2016).

### Molecular mode of action by which the active Cdt subunit, CdtB, induces toxicity

It is generally accepted that exposure of target cells to Cdt holotoxin leads to irreversible cell cycle arrest and cell death via apoptosis (Gelfanova et al., 1999; Cortes-Bratti et al., 2001; Shenker et al., 2001; Ohara et al., 2004). It should be noted that Cdt-induced cell cycle arrest typically involves arrest in the G2/M phase, although fibroblasts have been shown to undergo G1 arrest as well (Frisan et al., 2002). Another interesting phenomenon is that all cells are not susceptible to Cdt-induced toxicity (i.e., cell cycle arrest and apoptosis) and further that non-dividing cells such as non-activated primary human lymphocytes, macrophages, and mast cells do not undergo apoptosis in the presence of Cdt (Shenker et al., 2001, 2010, 2014; Smith and Bayles, 2006). These results are not necessarily surprising as many microbial toxins exhibit cell specificity. However, in most instances, cell specificity is attributed to toxin binding to cell receptors that are unique to the susceptible population(s) and thereby restrict toxin association and internalization. However, as discussed earlier, Cdt binding to cells and subsequent CdtB internalization is dependent upon association and binding to cholesterol, a ubiquitous membrane component. Consistent with this finding, we have also demonstrated that Cdt binding and CdtB internalization occurs in cells that are both Cdt-susceptible as well as Cdt-resistant (Shenker et al., 2016a). Thus, restricted susceptibility to Cdt intoxication must be governed by a mechanism independent of binding and subunit internalization. In this regard, we propose that cell susceptibility to Cdt is directly related to the toxin's molecular mode of action.

### Evidence that supports CdtB functioning as a DNase

The long-standing paradigm accounting for the molecular mechanism by which Cdt induces cell cycle arrest and ultimately apoptosis is based upon the active subunit's (CdtB) ability to function as a DNase (Lara-Tejero and Galán, 2000). The evidence in support of CdtB's ability to function as a DNase and thereby induce DNA damage has been the subject of numerous investigations and the focus of several recent review articles (Guerra et al., 2011b; Jinadasa et al., 2011; Grasso and Frisan, 2015; Taieb et al., 2016). Therefore, we only briefly summarize the evidence in support of this mode of action which is based upon the following three lines of investigation: (1) demonstration of *in vitro* DNase activity associated with CdtB; (2) nuclear localization of CdtB; and (3) activation of the DNA damage response (DDR).

**Figure 2:**
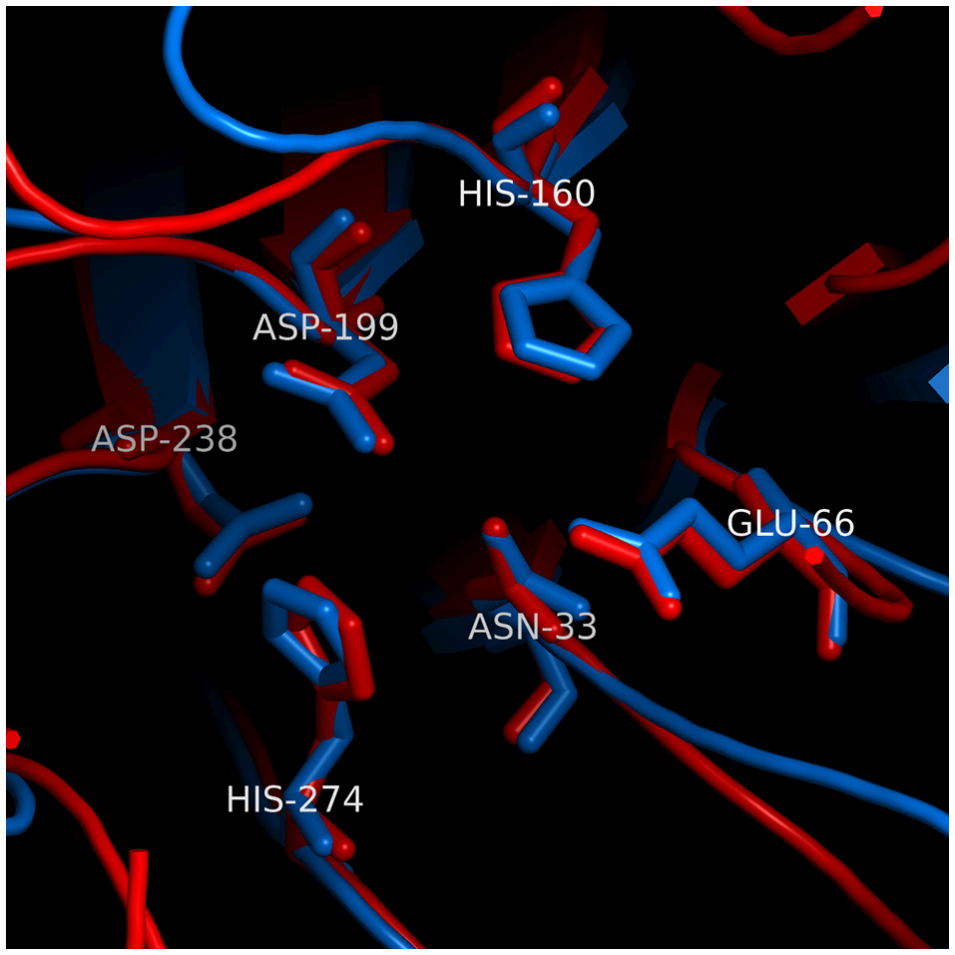
**Structural similarity between the active sites of CdtB and IP5P**. The superposition was based on conserved residues in the active sites of CdtB [blue; PDB code 2f2f_B (Yamada et al., 2006)] and IP5P [red; PDB code 1i9z_A (Tsujishita et al., 2001)]. The two sets of 6 conserved residues are aligned well, with an RMSD of 0.26 Å. Residue numbers correspond to CdtB structure. Parts of superimposed structures were clipped out of viewing plane for clarity.

Analysis of Cdt by X-ray crystallography demonstrates that CdtB exhibits structural similarity with a group of functionally unrelated metalloenzymes such as DNase I (Dlakić, 2000; Dlakic, 2001; Nesic et al., 2004; Yamada et al., 2006). Despite the lack of significant pairwise sequence identity, structural similarity was predicted on the basis of sensitive multiple sequence alignments as an extension of an earlier discovery of homology between the nucleases and bacterial sphingomyelinase (Matsuo et al., 1996). Analysis of CdtB indicates that its structure can be superimposed on eukaryote DNase I with an RMSD of 1.62 Å over 141 Cα atoms. A group of six conserved residues that are found in catalytic sites of DNase I and CdtB superimpose with an RMSD of 0.34 Å (see Figure 2 for the exact residue designation in CdtB). Direct evidence of DNase activity leading to strandbreaks and fragmentation of mammalian DNA has not been demonstrated to date. However, several investigators have shown that CdtB isolated from multiple bacteria is able to denature or relax supercoiled plasmid DNA *in vitro* (Elwell and Dreyfus, 2000a; Elwell et al., 2001; Mao and DiRienzo, 2002; Frisan et al., 2003; Nesic et al., 2004). Furthermore, mutations in putative catalytic amino acids have given rise to proteins with both reduced enzymatic activity and toxicity (Elwell and Dreyfus, 2000b; Lara-Tejero and Galán, 2000; Nesic et al., 2004; Hu and Stebbins, 2006; Shenker et al., 2007, 2016a).

CdtB-mediated DNA damage requires that the active Cdt subunit translocate from the cell surface to the nucleus where it is able to encounter its enzymatic substrate. Several lines of investigation have been employed to demonstrate nuclear localization of CdtB. For example, purified *E. coli* CdtB was observed in the nucleus of cells following introduction of the subunit by electroporation as well as after transfection and ectopic expression (McSweeney and Dreyfus, 2004); likewise, Lara-Tejero and Galán (2000) demonstrated transient expression of *C. jejuni* CdtB led to nuclear localization. Moreover, *E. coli* and *A. actinomycetemctomitans* CdtB mutants lacking nuclear localization signals failed to accumulate in the nucleus and intoxicate cells (Nishikubo et al., 2003; McSweeney and Dreyfus, 2004). In a more recent study, Damek-Poprawa et al. (2012) demonstrated nuclear localization of CdtB following exposure of Chinese hamster ovary cells (CHO-K1) to *A. actinomycetemcomitans* toxin.

While there is a paucity of direct *in vitro* evidence that CdtB-associated DNase activity contributes to fragmentation of mammalian DNA, ample indirect data is available demonstrating that Cdt-treated cells exhibit signs of damage to cellular DNA. Evidence in support of CdtB-mediated DNA damage is derived from experiments that demonstrate DDR activation in Cdt-treated cells. The DDR consists of multiple components such as sensors that detect strandbreaks, kinases and transcription factors that act as transducers and effector proteins that serve to delay cell cycle progression and promote DNA repair (Liu et al., 2016). For example, the presence of DNA strandbreaks has been shown to lead to formation of the MRN complex (Mre11, Rad50, and Nbs1) which in turn leads to recruitment and activation of the protein kinase, Ataxia telangiectasia mutated (ATM), and phosphorylation of the histone H2AX. Cdt's from *H. ducreyi, C. jejuni*, and *E. coli* have been shown to induce formation of the MRM complex and/or phosphorylation of H2AX in HeLa cells, fibroblasts, epithelial cells, and lymphocytes (Li et al., 2002; Hassane et al., 2003; Bielaszewska et al., 2005; Liyanage et al., 2010; Dixon et al., 2015). Additionally, Cdt-treated cells exhibit activation of the G2 checkpoint in a manner that is consistent with other agents known to induce DNA damage and activation of the DDR. Specifically, several investigators have demonstrated that Cdt treated cells exhibit increased levels of the active kinase Chk2 along with hyperphosphorylation of both (inactive) CDC25 phosphatase and the cyclin-dependent kinase CDK1 (Sert et al., 1999; Cortes-Bratti et al., 2001; Li et al., 2002; Sato et al., 2002; Yamamoto et al., 2004). In the absence of repair, activation of the G2 checkpoint should lead to apoptosis in addition to cell cycle arrest; an observation consistent with the known effects of Cdt. However, it has also been noted that if cells survive Cdt intoxication, CdtB-mediated DNA damage may contribute to genetic instability and thereby may promote carcinogenesis (Guidi et al., 2013).

It should be noted that in almost every instance, experiments demonstrating DNase activity, nuclear localization of CdtB as well as activation of the DDR were done under conditions of exposure to relatively high levels (microgram) of toxin as well as with “non-physiologic” routes of exposure. Interestingly, we and several other investigators have demonstrated that CdtB exhibits low DNase activity when compared with bovine DNase I (<0.01%, Elwell and Dreyfus, 2000a; Shenker et al., 2001; Haghjoo and Galán, 2004; Nesic et al., 2004) perhaps explaining the requirement for employing high toxin doses. Additionally, it should be noted that several of these studies failed to differentiate between DNA damage due to direct CdtB associated DNase activity and that due to indirect effects resulting from caspase mediated activation of endogenous DNase; the latter is supported by our own studies on lymphocytes and HeLa cells (Gelfanova et al., 1999; Shenker et al., 2001; Nalbant et al., 2003; Thelastam and Frisan, 2004). Specifically, inhibition of the apoptotic cascade by over expression of Bcl-2 or with the use of a caspase-3 inhibitor results in suppression of the DDR with no effect on Cdt-induced cell cycle arrest. Moreover, under conditions optimized to expose cells to the minimal amount of Cdt necessary to induce maximal G2 arrest within 24 h (and apoptosis at 48 h), we were unable to demonstrate DDR activation in lymphoid cells at 4 h. DDR activation could only be induced following longer exposures to higher Cdt concentrations for both lymphocytes and HeLa cells (Shenker et al., 2016a). These results collectively argue for DDR activation, at least in lymphocytes, to be the result of late events, and likely associated with enzymatic degradation of DNA due to activation of the apoptotic cascade.

### Evidence that CdtB functions as a phosphatidylinositol-3,4,5-triphosphate (PiP3) phosphatase

As noted above, the bias in the literature supporting a role for CdtB associated DNase activity as the underlying mode of action function was based, in part, on structural homology with DNase I. This relationship obscures the fact that CdtB's protein fold and reaction mechanism are also shared with a number of metalloenzymes (Dlakić, 2000; Dlakic, 2001). CdtB structure can also be superimposed on inositol polyphosphate-5-phosphatase (IP5P), another member of this superfamily, with an RMSD of 1.63 Å over 121 Cα atoms, which is similar to that observed with DNase I. The six conserved residues in the catalytic site of CdtB (Yamada et al., 2006) and IP5P (Tsujishita et al., 2001) superimpose with an RMSD of 0.26 Å (Figure 2), which is slightly better than the alignment of those same residues between CdtB and DNase I. This is not surprising since CdtB, IP5P, and DNase are all phosphoesterases (Dlakić, 2000; Dlakic, 2001); the specific function of each of these enzymes is largely dependent upon accommodation of substrates within their respective active site (Shenker et al., 2016a). Over the past several years, we have generated evidence for a new paradigm to account for the molecular mode of action by which *A. actinomycetemcomitans* CdtB intoxicates cells. Central to this mechanism is CdtB's ability to function as a lipid phosphatase similar to IP5P. As a result, Cdt is then able to perturb the critically important signaling pathway, the phosphoinositide 3-kinase (PI-3K) signaling pathway, that regulates cell survival, growth and a variety of functions that are cell type specific.

PIP3 is now recognized for its critical role in regulating cell growth, proliferation, and survival, among others (Kraub and Haucke, 2007; Sasaki et al., 2007; Buckler et al., 2008; Huang and Sauer, 2010). To be effective as a second messenger, PIP3 is maintained at low baseline levels which may increase rapidly in response to signals that typically originate at the plasma membrane leading to PI-3K activation. PIP3 levels are tightly regulated by three degradative enzymes: PTEN, SHIP1, and SHIP2 (Krystal, 2000; March and Ravichandran, 2002; Seminario and Wange, 2003). PTEN (*p*tase and *t*ensin homolog deleted on chromosome *ten*), hydrolyzes PIP3 to PI-4,5-P_2_. SHIP1 (*SH*2-containing *i*nositol *p*hosphatase) and SHIP2 are inositol 5-phosphatases (IP5P); SHIP2 is ubiquitously expressed while SHIP1 is found in a limited subset of cells which includes immune cells. Both SHIP enzymes hydrolyze PIP3 to PI-3,4-P_2_ and inositol 1,3,4,5-tetrakisphosphate to inositol 1,3,4 triphosphate. The PI-3K/PIP3/Akt signaling pathway is regulated collectively by the action of these synthetic and degradative enzymes. Of particular relevance to Cdt toxicity, we have shown that the active Cdt subunit, CdtB, exhibits potent PIP3 phosphatase activity similar to that of SHIP.

In order to first establish a clear association between Cdt-mediated toxicity and CdtB's ability to function as a PIP3 phosphatase, an extensive analysis of CdtB mutants was carried out. Mutants were designed to target residues predicted to be part of the active site and/or involved in substrate binding. Additional residues were targeted because they are conserved in both CdtB and IP5P but not in DNase I; other residues were targeted because they are conserved in IP5P and DNase I but not in CdtB. Analyses of this array of CdtB mutants clearly demonstrate a strong correlation between Cdt toxicity and retention of CdtB PIP3 phosphatase (Shenker et al., 2016a). Another key finding was that some mutants exhibited improved DNase activity; however this increase in enzymatic activity did not correlate with increased cytotoxicity suggesting that DNase function of CdtB is secondary to the lipid phosphatase function. This is consistent with the notion that a single enzyme is unlikely able to equally accommodate two substrates, such as DNA and lipid phosphates, as they significantly differ in both size and overall structure. Nonetheless, both molecules contain phosphate esters and are hydrolyzed, to some extent, by multiple enzymes such as those that comprise the superfamily that includes both CdtB and DNase I (Dlakić, 2000).

### CdtB induces PI-3K signaling blockade

Mutation analysis discussed above is consistent with the notion that CdtB perturbs the PI-3K/PIP3/Akt signaling pathway (Shenker et al., 2007, 2014; Boesze-Battaglia et al., 2015). More recent studies demonstrate that CdtB perturbs this signaling pathway within a few hours of exposure to Cdt; these changes include a decline in PIP3 levels and a concomitant increase in PI-3,4-P2, the enzymatic product of PIP3 degradation. Similar changes were observed for human peripheral blood mononuclear cells (HPBMC) activated by mitogen (Shenker et al., 2016a).

PI-3K activation leads to not only PIP3 production, but also phosphorylation of the critical downstream kinase, Akt; phosphorylation of Akt (pAkt) leads to its activation. Thus, it is noteworthy that Shenker et al. (2016a) observed that Cdt-treated lymphocytes not only exhibit PIP3 depletion, but also significant decreases in pAkt; this change in phosphorylation status was accompanied by a concomitant decrease in its kinase activity. A downstream target of pAkt is another kinase, GSK3β. Activation of Akt typically leads to phosphorylation and inactivation of GSK3β (pGSK3β). Indeed toxin treated cells were found to exhibit a reduction in the phosphorylation of GSK3β; reduced pGSK3β was accompanied by an increase in its kinase activity. This observation is critical since GSK3β, initially discovered as an inhibitor of glycogen synthase, has recently been implicated in the regulation of cell functions associated with inflammation, cell proliferation, and apoptosis, among others (Luo, 2009; Rayasam et al., 2009). It is noteworthy that pre-treatment of cells with GSK3β inhibitors reduced their susceptibility to Cdt intoxication; these results are consistent with a mode of action involving Cdt-mediated PI-3K signaling blockade, suppression of cell growth and promotion of cell death (Buckler et al., 2008; Yuan and Cantley, 2008; Huang and Sauer, 2010).

CdtB associated lipid phosphatase activity and PI-3K blockade also contributes to toxicity in non-lymphoid cells. Under conditions optimized to induce cell cycle arrest at 24 h, Shenker et al. (2016a) observed that Cdt holotoxin containing CdtB^WT^ induced phosphorylation of H2AX at 16 h. These findings are consistent with results reported by other investigators following exposure to significantly higher concentrations of toxin (Li et al., 2002; Fahrer et al., 2014). In contrast, substitution of CdtB wildtype with a CdtB mutant that was shown to retain phosphatase and no DNase activity (CdtB^A163R^), failed to induce phosphorylation of H2AX; this mutant did induce G2 arrest as observed with CdtB^WT^. These results clearly demonstrate that HeLa cells undergo Cdt-induced G2 arrest in the absence of CdtB-associated DNase activity and detectable DDR activation. It is noteworthy that these results are consistent with those of Sert et al. (1999) who reported that toxin-treated HeLa cells underwent G2 arrest in a manner that was independent of DNA damage. Interestingly, Shenker et al. (2016a) demonstrated that toxin comprised of CdtB mutants which retain DNase activity but deficient in PIP3 phosphatase activity lost the ability to induce HeLa cells to undergo G2 arrest; H2AX phosphorylation was also not observed in these cells. Cdt induced PI-3K blockade was further corroborated by the observation that GSK3β inhibitors could block toxin-induced G2 arrest in HeLa cells as described above for lymphoid cells (Shenker et al., 2016a).

Shenker et al. (2016a) did not rule out a possible role for DNase activity in mediating some aspects of Cdt toxicity. First, it is important to emphasize that PIP3 phosphatase activity has only been demonstrated for *A. actinomycetemcomitans* Cdt; it is possible that other Cdts are more dependent upon DNase activity to induce toxicity. Second, cells that exhibit resistance to Cdt and require exposure to high doses of Cdt in order to become intoxicated may indeed be responding to cellular alterations that are the result of CdtB associated DNase activity (Deng et al., 2001; Li et al., 2002; Guerra et al., 2011a). One argument against a primary role for Cdt-mediated PI-3K blockade is the observation that *Saccharomyces cerevisiae* are susceptible to Cdt-induced cell cycle arrest (Hassane et al., 2001; Matangkasombut et al., 2010); these cells do not synthesize PIP3. Nonetheless, *S. cerevisiae* do synthesize a myriad of phosphatidylinositol mono and di-phosphates; these phosphoinositides regulate many cell functions including growth and survival (Gardocki et al., 2005; Strahl and Thorner, 2007). To date there is no evidence demonstrating whether CdtB is able to alter any of these moieties in *S. cerevisiae*. Another area of controversy involves the link between the PI-3K pathway and regulation of cell size. This linkage lead Frisan et al. (2003) to suggest a critical role for PI-3K signaling in the development of cellular distension, a hallmark of Cdt toxicity; however, these authors did not provide evidence for PI-3K activation. It should also be noted that others have shown that there is no linkage between PI-3K signaling, cell size regulation and cell cycle arrest (Lee et al., 2007).

## Evidence that Cdt expression is critical to disease

As noted in Table 1, Cdts have been identified in a wide range of pathogenic species that infect a number of host tissues often in association with the development of significant inflammation. To date there is limited evidence that directly implicates Cdts as a virulence factor responsible for the molecular pathogenesis of disease in response to infection by Cdt-producing pathogens. Most evidence is circumstantial and based upon the presence of Cdt genes and/or protein in clinical isolates from pathogenic bacterial strains (summarized in Table 1). The identification of these Cdt-producing pathogens was initially based upon evaluation of bacterial extracts or supernatants for the presence of toxic activity and in some instances by genotyping for the presence of Cdt genes. More recently, several investigators have provided direct evidence suggesting that the pathogenicity of some pathogens is indeed linked to their ability to produce biologically active Cdt and thereby induce cytotoxic effects on host tissue. The evidence suggesting that Cdt is a crucial virulence factor is summarized in this section.

### *E. coli* Cdt

The presence of Cdt in clinical isolates of *E. coli* 0128 and 055:K59:H4 was first reported in the 1980s' in association with gastroenteritis (Anderson et al., 1987; Johnson and Lior, 1988b). Although a causal relationship was not established, the authors suggested that Cdt may have encephalopathic potential based on clinical presentation of disease in a 3-year old patient. Since these initial observations, there have been numerous reports demonstrating the presence of Cdt in significant portions of clinical isolates of enteropathogenic *E. coli* (reviewed in Smith and Bayles, 2006); five cytolethal distending toxin sequence variants were detected in these *E. coli* strains (Clarke, 2001; Pickett et al., 2004; Hinenoya et al., 2009) and more recently, Shiga toxin-producing *E. coli* (Clarke, 2001; Janka et al., 2003; Bielaszewska et al., 2004). With respect to the latter, the authors suggested that Cdt-positive strains were significantly associated with clinical symptoms such as hemolytic uremic syndrome and watery diarrhea. In other studies, a putative role for Cdt in EPEC infections could not absolutely be determined because of the co-expression of other virulence factors by clinical isolates or in some instances, the low incidence rate of Cdt among isolates (Albert et al., 1996; Okeke et al., 2000). Additionally, Cdt-producing *E. coli* from serogroup 086:K61 were reported as a possible cause of mortality in birds (Foster et al., 1998). Culture supernatants from several of these strains were also tested on mammalian cell cultures and found to cause distinct morphological changes, cell cycle arrest and cell death implicating Cdt effects on vascular endothelium as well as on epithelial cells of the urinary and/or gastrointestinal tracts. The significance of these *in vitro* findings in the context of virulence and disease pathogenesis is discussed in the next section.

### *Shigella* spp. Cdt

The production of Cdt was also described in *Shigella dysenteriae* type 2 and *S. boydii* type 7 strains. Similar to *E. coli* Cdts, *Shigella* Cdt was implicated in inflammatory responses of the gut (Johnson and Lior, 1988b). Okuda et al. (1997) provided the first *in vivo* experimental evidence of the diarrheagenicity of the *S. dysenteriae* Cdt in a suckling mouse model. Cdt-containing culture supernatant and partially purified Cdt preparations induced watery diarrhea. The incidence of loose/watery feces in mice subjected to Cdt was much higher as compared to animals given supernatants from Cdt-negative bacteria strains. The correlation between the amount of Cdt in the preparations and the diarrhea score, expressed as percentage of the diarrhea-positive animals in test groups, demonstrated the diarrheagenicity potential of Cdt.

### *Campylobacter* spp. Cdt

A cytotoxic factor, later defined as Cdt, was detected in culture filtrates of numerous *C. jejuni, C. coli, and C. laridis* strains recognized as enteric pathogens in human diarrheal disease as well as *C. fetus* subs. fetus associated with bovine abortion. Exposure of epithelial cells to *Campylobacter* spp. Cdt resulted in cell elongation, distention, and subsequent death within 96–120 h. Homologous rabbit antitoxin neutralized Cdt activity (Johnson and Lior, 1988a; Mizuno et al., 1994). *C. jejuni* Cdt-induced HeLa and Caco-2 cell death following cell cycle arrest in early G2/M phase (Whitehouse et al., 1998; Jain et al., 2009).

Cell-free preparations from *C. jejuni* strains 81–176 and NCTC 11168 containing the CdtB subunit inactivated by insertional mutation had either much lower toxicity or were completely inactive in HeLa cell cytotoxicity assays. Following intragastric inoculation of adult severe immunocompromised (SCID) mice, *C. jejuni* CdtB-negative mutants showed similar intestinal colonization but greatly reduced invasiveness into blood, liver, and spleen as compared to isogenic wild-type strains. Moreover, descending colons of SCID mice were damaged in the presence of the active toxin (Purdy et al., 2000). Wild-type *C. jejuni* strain but not its Cdt-lacking isogenic mutant was capable of causing more progressive gastritis and proximal duodenitis in a mouse oral gavage model (Fox et al., 2004a) and in the suckling mouse model (Jain et al., 2008). It has been shown that Cdt-producing *C. jejuni* strains exhibit greater adherence to and invasion of epithelial cells than mutants lacking Cdt activity. Fox et al. (2004a) reported that production of Cdt may facilitate bacterial escape of immune surveillance by inducing cell cycle arrest and apoptosis in lymphocytes. Unlike the wild-type, *C. jejuni* mutant lacking Cdt activity was eliminated from immunocompetent mice; the bacteria persisted in NF-κB-deficient mice.

Cdt was also detected in sonicates prepared from multiple *Helicobacter hepaticus* strains, closely related to *Campylobacter* species. These toxin preparations were shown to display cytopathic effects on HeLa cells similar to *C. jejuni* Cdt (Young et al., 2000b). The production of biologically active Cdt, as confirmed by *in vitro* assays using HeLa, Caco-2, and HCA-7 cells, was also reported in a number of other *Helicobacter* species, including human pathogens *H. pullorum* (Young et al., 2000b; Varon et al., 2014), *H. canis* (Chien et al., 2000), and *H. cinaedi* (Taylor et al., 2003). Interestingly, to date Cdt is the only known virulence factor present in *H. hepaticus* (Liyanage et al., 2010). Furthermore, a *H. hepaticus* isogenic mutant of the Cdt gene cluster had diminished capacity to induce lesions in the gut of C57BL/6 interleukin 10(−/−) mice in a model of inflammatory bowel disease. The animals infected with wild-type *H. hepaticus* developed severe mucosal hyperplasia accompanied by inflammation of the lamina propria, while the mice colonized with Cdt mutant exhibited only mild, patchy mucosal inflammation (Young et al., 2004; Pratt et al., 2006). Additionally, Ge et al. (2005) showed that the presence of biologically active Cdt is crucial for persistent colonization of *H. hepaticus* in cecum, colon and jejunum of out bread Swiss Webster mice.

### *H. ducreyi* Cdt

*H. ducreyi* was shown to secrete a soluble cytotoxin that leads to the death of both HeLa and Hep-2 cells; it was further proposed that this toxin was a putative virulence factor contributing to the pathogenicity of this bacterium (Lagergård, 1992; Purvén and Lagergard, 1992; Lagergård and Purven, 1993; Purvén et al., 1995). This virulence factor was later identified as a product of the Cdt gene cluster. It should also be noted that in several studies, 64–89% of clinical isolates tested contained the Cdt genes (Purvén et al., 1995; Frisk et al., 2001). Culture supernatant obtained from a strain of *H. ducreyi* lacking the Cdt genes was unable to kill epithelial cells (Cope et al., 1997; Stevens et al., 1999). Expression of all three *H. ducreyi* recombinant Cdt subunits in *E. coli* was necessary to exert toxic effects on HEp-2 cell cultures (Frisk et al., 2001). Cortes-Bratti and co-investigators (Cortes-Bratti et al., 1999) demonstrated cell cycle arrest and epithelial cell death in the presence of *H. ducreyi* Cdt suggesting that the toxin may contribute to the development and chronic nature of the ulcerative lesions associated with chancroid.

Further, *in vitro* and *in vivo* studies provided compelling evidence for a role of Cdt as a bacterial virulence factor. Hemolysin-deficient *H. ducreyi* mutant producing Cdt elicited similar inhibitory and apoptotic effects on peripheral blood mononuclear cells and Jurkat cells as its isogenic parent (Gelfanova et al., 1999). However, when the *H. ducreyi* CdtA, CdtB, and/or CdtC subunit(s) were deleted, the toxin preparations exhibited lower (in case of cdtA mutant) or no cytotoxicity for HeLa cells. Surprisingly, no change in virulence of *H. ducreyi* Cdt mutants was observed in a temperature-dependent rabbit model (Lewis et al., 2001). Isogenic *H. ducreyi* CdtC mutant and its parent clone were also shown to be similarly virulent in pustule formation in the early stages of chancroid in human volunteers (Young et al., 2001). Nonetheless, purified *H. ducreyi* CdtA, CdtB, and CdtC, injected as a complex in rabbit skin, caused dose-dependent erythema and edema as well as inflammatory cell infiltration and dilatation of blood vessels. Individual protein subunits had no pathological effect on rabbit skin following intradermal injection (Wising et al., 2002).

### *A. actinomycetemcomitans* Cdt

Cdt with amino acid sequence similar to *H. ducreyi* Cdt was also detected in *Actinobacillus* (currently *Aggregatibacter*) *actinomycetemcomitans* culture supernatants (Sugai et al., 1998; Ahmed et al., 2001; Yamano et al., 2003). All *A. actinomycetemcomitans* members of the restriction fragment length polymorphism (RFLP) group II cluster (DiRienzo and McKay, 1994), which was shown to be associated with the conversion in young children from periodontal health to localized aggressive periodontitis (LAP; Mayer et al., 1999), contained a complete *cdt* gene locus (Mayer et al., 1999). The toxin was shown to cause cell distention and cell cycle arrest in HeLa cells. The combination of all three recombinant *A. actinomycetemcomitans* Cdt protein subunits expressed in *E. coli* was necessary to induce HEp-2 cell cycle arrest (Akifusa et al., 2001). Exposure of epithelial cells to individual subunits failed to block cell cycle progression.

Human lymphocytes were found to be exquisitely sensitive to *A. actinomycetemcomitans* Cdt; small quantities (picogram) of toxin were capable of inducing cell cycle arrest and apoptosis within 24 and 48 h, respectively (Shenker et al., 2001, 2004). Similar results were reported by Nalbant et al. (2003). In contrast, to the effect of Cdt on proliferating lymphoid cells, the toxin failed to kill human monocytes and macrophages (Shenker et al., 2014, 2015). Instead, exposure to Cdt resulted in the synthesis and release of pro-inflammatory cytokines. Likewise, Akifusa (Akifusa et al., 2001) demonstrated that Cdt induced IL-1β, IL-6, and IL-8 cytokine production in human peripheral blood mononuclear cells (PBMCs).

The importance of Cdt as a potential *A. actinomycetemcomitans* virulence factor was underscored by its role in the induction of receptor activator of NF-κB ligand (RANKL) expression in human gingival fibroblasts and periodontal ligament cells. This is a critical finding as RANKL expression is associated with osteoclast differentiation and thereby contributes to regulation of bone resorption. Thus, the possibility exists that Cdt-induced expression of RANKL may be involved in the pathogenesis of periodontitis (Mogi, 2004). Purified recombinant *H. ducreyi* Cdt also induces RANKL expression in osteoclasts. Furthermore, pretreatment of *A. actinomycetemcomitans* wild-type extract with Cdt neutralizing antibody abolished RANKL expression (Belibasakis et al., 2005a). The detrimental effect of the *A. actinomycetemcomitans* Cdt on periodontal tissues was confirmed using an *in vivo* rat model. Topical application of Cdt to the gingival sulcus of rats resulted in junctional epithelial tissue abrasion. Interestingly, animals treated with both wild-type and mutated Cdt exhibited neutrophil infiltration. However, epithelial cell damage occurred only in rats treated with wild-type Cdt (Ohara et al., 2011). Damaging effects of wild-type *A. actinomycetemcomitans* Cdt on gingival epithelia were also detected in rat and human gingival explants (Damek-Poprawa et al., 2011, 2013).

### *Salmonella enterica* subsp. enterica serotype typhi (*S*. typhi) Cdt

As noted earlier, the CdtB gene was also detected in *S*. Typhi (Parkhill et al., 2001; Haghjoo and Galán, 2004). While other Salmonella serovars cause self-limiting gastroenteritis, *S*. Typhi causes persistent and life-threatening systemic disease. *S*. Typhi produces a unique toxin, typhoid toxin, in which the active (A) unit is comprised of two subunits: CdtB and PltA, a homolog of the active subunit of pertussis toxin. Song et al. (2013) demonstrated that systemic administration of typhoid toxin into mice induced many of the symptoms commonly observed in the early stages of typhoid fever. Also, a non-typhoidal *Salmonella enterica* serovar Javiana was found to express Cdt; this organism is associated with gastrointestinal illnesses in humans, cattle, birds, and reptiles (Mezal et al., 2014). Biologically active *S*. Javiana Cdt was shown to increase invasiveness of the bacteria in HeLa cell cultures. These observations suggest a role for CdtB as an important virulence attribute of *Salmonella*. spp. (Mezal et al., 2014).

## Cdt functions as a tri-perditious virulence factor by targeting host defenses

The role of a virulence factor is defined, in part, by key features such as its ability to associate with a specific cell(s) and its molecular mode of action. Taking into account that Cdt-producing bacteria typically infect mucocutaneous tissue, we now propose that Cdt contributes to microbial virulence by virtue of its ability to function as a tri-perditious toxin as it targets host defenses at three levels (see Figure 1): (1) promotion of infection by altering barrier protection through modulation of epithelial cell growth and survival, (2) promotion of inflammatory responses through activation of the inflammasome leading to cytokine maturation and release, and (3) impairment of acquired immunity and as a consequence diminished immune elimination by inhibiting T- and B-cell proliferation and survival. In this section, we discuss the evidence that Cdt targets these three host systems.

Cdt is capable of intoxicating a wide range cells and/or cell lines, regardless of microbial source. Intoxication of proliferating cell populations is characterized by distinct morphological alterations, inhibition of cell growth most commonly associated with G2 arrest of the cell cycle and ultimately cell death resulting from activation of the apoptotic cascade (Jinadasa et al., 2011). In contrast, non-proliferating cells, such as macrophages, mast cells, and osteoclasts are resistant to Cdt-induced cell death; however, these cells are susceptible to Cdt-mediated alterations in cell function. The ability of a single toxin to affect multiple cell types is consistent with current understanding of the underlying mechanism of Cdt-host cell interactions which is dependent upon binding to cholesterol, a ubiquitous membrane component, as opposed to hijacking a unique receptor associated with a specific cell type. As noted earlier, Cdt from *C. jejuni* and *S*. Typhi are not dependent upon cholesterol binding. Moreover, the ability to intoxicate multiple cell types leading to different outcomes is also consistent with the proposed paradigm shift in which CdtB functions as a PIP3 phosphatase and targets the key PI-3K signaling pathway. As PI-3K is a signaling pathway commonly used by most cells to regulate a variety of functions, blockade of this pathway will lead to alterations involving a range of cell activities which are ultimately defined by the unique programming of each individual cell type. It should also be noted that hijacking of the PI-3K signaling cascade is also consistent with Cdt's association with cholesterol in the context of lipid rafts. These membrane moieties have been shown to serve as platforms for a number of signaling cascades in which substrates and critical components accumulate. It should also be noted that lipid rafts and sphingomyelin-controlled protein scaffolds are important for lipid signaling in cells; thus the observations of Carette et al. (2009) are critical as they demonstrate that cells deficient in sphingomyelin synthase are also resistant to Cdt intoxication. These results further suggest that lipid rafts are important for CdtB entry into cells.

### Cdt impairs epithelial barriers

Epithelial cells constitute the mechanical barrier between the host and an environment often teeming with microbes; thus, perturbation of protective epithelial layers exposes underlying tissues to bacteria and their products. As noted above, Cdt-producing bacteria identified to date are pathogens that exhibit an affinity to colonize mucocutaneous tissue such as oral, gastrointestinal, urinary, and respiratory tracts (Jinadasa et al., 2011). Moreover, the subsequent development of disease in these mucocutaneous niches is clearly the result of sustained infection and inflammation. For example, Cdt-producing *Campylobacter, Escherichia, Helicobacter, Shigella, and Salmonella* species account for a majority of food- and water-borne infectious diarrheal illnesses in humans (Tauxe, 1997; Ge et al., 2008; Jinadasa et al., 2011) and animals (Black et al., 1988; Dassanayake et al., 2005a; Johnson et al., 2007). Epithelial cells intoxicated with Cdt undergo G2 arrest leading to apoptosis (Ceelen et al., 2006a; Smith and Bayles, 2006; Liyanage et al., 2010, 2686/id). Cdt is also responsible for decreased intestinal epithelial cell adherence that results from alteration of actin cytoskeleton and disturbance of focal adhesion and microtubule network (Schulze et al., 1998; Varon et al., 2014).

In the oral cavity, highly proliferative primary human gingival epithelial cells (HGEC) are sensitive to the toxin produced by *A. actinomycetemcomitans* Cdt and exhibit a response similar to that observed with a Cdt-treated immortalized human oral vestibular epithelial cell line (GMSM-K; Kang et al., 2005; Kanno et al., 2005; Damek-Poprawa et al., 2011). Following intoxication, HGEC undergo rapid G2 arrest. As noted earlier, *ex vivo* experiments demonstrated that Cdt altered the integrity of the gingival epithelial barrier; damage included detachment of the keratinized outer layer, distention of spinous and basal cells in the oral epithelium, disruption of rete pegs, and dissolution of cell junctions (Damek-Poprawa et al., 2013). Likewise, the genital pathogen *H. ducreyi*, a well-established cause of a sexually transmitted chancroid (Abeck et al., 1997), has recently emerged as a major etiologic agent in chronic limb ulceration similar to yaws in the Pacific region and Africa. Infection with *H. ducreyi* occurred following trauma to the epithelial layer and Cdt-induced epithelial cell death was a contributing factor in slow healing of lesions (Lewis and Mitja, 2016).

### Cdt impairs acquired immunity

To date there have been few studies that assess immunity to Cdt following natural or experimental exposure. Nonetheless, studies on *A. actinomycetemcomitans* Cdt, as well as the closely related Cdt from *H. ducreyi*, indicate that anti-Cdt responses in patients with LAP or chancroid who do harbor the respective pathogens, generally produce low levels of antibody. Moreover, in most instances where antibodies were detected, the incidence and potency of Cdt-neutralizing antibodies was low (Lagergård and Purven, 1993; Xynogala et al., 2009; Ando et al., 2010; Lundqvist et al., 2010). The authors concluded that either the holotoxin is not immunogenic or its ability to function as an immunotoxin interferes with the host's ability to respond to it as an immunogen. These authors also suggested that the inability to neutralize Cdt likely contributes to failure of the host's immune system to eliminate the microbe and thereby limit disease. Anecdotally, we have assessed >20 LAP patient sera and 20% were capable of toxin neutralization (unpublished data) and further, we have also generated >100 anti-Cdt monoclonal antibodies and found that only five were capable of toxin neutralization. In contrast, Wising et al. (2002) has demonstrated that immunization of rabbits with a toxoid prepared from Cdt results in a robust immune response capable of toxin neutralization. These results further support the notion the Cdt functions as an immunotoxin; although these latter observations could also be the result of novel epitopes becoming available when challenged with the toxoid. Finally, it should be noted that *S*. Typhi establishes persistent infection; this is an observation consistent with the action of an immunosuppressive toxin. As noted above, typhoid toxin is comprised of active subunits of both pertussis toxin and Cdt. It has been suggested that the immunomodulatory activity of the CdtB component of the typhoid toxin may be a contributing factor to this persistent infection (Haghjoo and Galán, 2004). Moreover, injection of typhoid toxin into mice induces many of the early symptoms associated with typhoid fever as well as a significant reduction in immune cells (Song et al., 2013).

Consistent with the discussion above, it is now well-established that lymphocytes, both B and T, are among the most susceptible cells to Cdt. We first demonstrated that treatment of Jurkat cells and mitogen activated HPBMC with low levels (picogram) of *A. actinomycetemcomitans* Cdt resulted in inhibition of proliferation and G2 arrest within 24 hr; interestingly, these cells did not exhibit cellular distension as has been characterized for other cells (Shenker et al., 1999, 2000). Exposure to these same doses of Cdt resulted in apoptosis after 48 h. Nalbant et al. (2003) observed that Cdt holotoxin could also inhibit HPBMC proliferation; mutants lacking the CdtB subunit were deficient in this inhibitory effect. Likewise, Gelfanova et al. (1999) demonstrated that *H. ducreyi* Cdt also inhibited proliferation and induced apoptosis in these same cell populations. In 2001, Mooney et al. (2001) demonstrated that lysates of *C. upsaliensis* contained Cdt which could also induce G2 arrest in human T-cells. These findings have been confirmed by other investigators as well (Cortes-Bratti et al., 2001; Svensson et al., 2001; Sato et al., 2002). Xu et al. (2004) also demonstrated that monocyte-derived dendritic cells treated with *H. ducreyi* Cdt did not exhibit cytokine responses or support T-cell proliferation; these findings lead these authors to propose that the toxin not only impairs lymphocyte proliferation and survival, but may also interfere with early stages of the developing immune response.

### Cdt promotes pro-inflammatory macrophage responses

Innate immunity represents the first line of defense against microbial pathogens. A key component in the activation of innate immune responses involves Toll-like receptors (TLRs); recognition of microbial products by TLRs leads to the production of pro-inflammatory cytokines. In general, this pro-inflammatory response protects the host. However, excessive or prolonged release of pro-inflammatory cytokines may eventually turn this protective function to one that contributes to overt tissue damage. Conversion from a protective to destructive role is of particular relevance as many diseases associated with Cdt-producing pathogens involve persistent infection along with a robust inflammatory response. Periodontal disease, for example, is considered to be a bacterial-induced inflammatory disorder culminating in the destruction of tooth-supporting tissues. One form of periodontal disease, LAP, is associated with infection by Cdt-producing *A. actinomycetemcomitans* (Xynogala et al., 2009). Experimental and clinical investigations into the pathogenesis of LAP demonstrate the presence of high levels of pro-inflammatory cytokines within the periodontium (Ando et al., 2010). In addition to tissue damage which ultimately contributes to tooth loss, the degraded proteins along with hemin may serve the nutritional needs of the bacteria to further promote their growth and survival (Hajishengallis et al., 2012).

As noted above, there is general consensus that macrophage survival is not altered by Cdts (Svensson et al., 2001; Shenker et al., 2014); however, macrophages are indeed susceptible to Cdt intoxication. Cdt has been shown to perturb PI-3K signaling in human monocytes and macrophages derived from the THP-1 cell line. Specifically, Cdt treatment resulted in decreases in Akt and GSK3β phosphorylation; concomitant alterations in kinase activity was observed with lymphocytes (Shenker et al., 2014). Exposure to Cdt did not lead to macrophage death; however, the toxin did induce a pro-inflammatory cytokine response characterized by increased expression and release of IL-1β, IL-18, TNFα, and IL-6. Interestingly, Cdt also augmented the pro-inflammatory cytokine response stimulated by TLR-2, -3, or -4 agonists. These findings are consistent with CdtB-mediated PI-3K bockade, GSK3β activation and NF-κB activation; the latter is known to lead to increased expression of pro-inflammatory cytokines (Arbibe et al., 2000; Guha and Mackman, 2002; Hazeki et al., 2007; Molnarfi et al., 2007; Brown et al., 2010; Antoniv and Ivashkiv, 2011).

Release of mature IL-1β and IL-18 is known to be dependent upon caspase-1 activation; maturation of caspase-1 is dependent upon the activation of inflammasomes. Indeed, the Cdt-induced pro-inflammatory response was also observed to be dependent upon activation of the NLRP3 inflammasome. Shenker et al. (2015) have demonstrated Cdt-mediated inflammasome activation is dependent upon toxin-induced increases in extracellular ATP, activation of the purinergic receptor, P2X_7_ and K^+^ efflux. It should also be noted that Belibasakis and Johansson (2012) have shown that treatment of human monocytes with *A. actinomycetemcomitans* leads to increased expression of NLRP3; however, they reported that this effect was not dependent upon Cdt. The authors did not indicate if inflammasome activation occurred in toxin treated cells or whether mature cytokine was secreted.

## Summary

In this review, we have addressed a key issue relevant to the role of Cdt as a virulence factor. Many cells have been shown to be susceptible to Cdt intoxication; however, it should be noted that cells do exhibit differential susceptibility to Cdt in terms of absolute sensitivity. Specifically, lymphocytes and possibly epithelial cells represent the highly sensitive side of the spectrum requiring picogram to nanogram quantities of Cdt, while HeLa cells and fibroblasts represent the least sensitive side typically requiring microgram quantities of toxin; macrophages exemplify cells of moderate sensitivity requiring nanogram quantities. We propose that differential cell sensitivity to Cdt reflects variance in the dependence of cells on PI-3K signaling for cell survival, growth and/or cell function. Moreover, toxin susceptibility is likely influenced by baseline levels of intracellular PIP3 which reflect the state of activity of this signaling pathway. Indeed, we have previously demonstrated that lymphoid susceptibility to Cdt correlates with baseline PIP3 levels; lymphoid cells with high levels of PIP3 and active PI-3K signaling exhibit heightened susceptibility to Cdt vs. those with low levels of the lipid second messenger and relatively inactive signaling exhibited reduced sensitivity (Shenker et al., 2016b). In conclusion, we propose that Cdt functions as a tri-perditious virulence factor as it targets host defenses in three ways: (1) altering barrier protection (via epithelial toxicity) thereby promoting infection: (2) impairment of acquired immunity (via lymphocyte toxicity) reducing immune elimination; and (3) promotion of inflammation (cytokine expression by macrophages). Cdt's ability to mount a three-pronged attack against host defenses is facilitated by its unique ability to utilize cholesterol, a ubiquitous moiety to bind to plasma membranes and to target cell homeostasis and function through a signaling pathway common to all cells. It is important to point out that lipid phosphatase activity has only been demonstrated for CdtB from *A. actinomycetemcomitans*. However, based upon sequence homology, we believe that CdtB from *H. ducreyi* would exhibit similar activity. Moreover, since all CdtB's analyzed to date exhibit relatively poor DNase activity, it is reasonable to expect that toxin produced by other bacteria will act through a similar mode of action. Thus, we anticipate that future studies will demonstrate that CdtB's from other Cdt producing pathogens will exhibit robust PIP3 phosphatase activity similar to that of *A. actinomycetemomitans* CdtB.

## Author contributions

The ideas and concepts presented in this review are the synthesis and compilation of the ideas developed from all four authors. Each author contributed text and/or figures to the manuscript. Each author had significant input into this manuscript.

### Conflict of interest statement

The authors declare that the research was conducted in the absence of any commercial or financial relationships that could be construed as a potential conflict of interest.
